# Profile of SARS-CoV-2/HIV-1 coinfection in patients from the extreme south of Brazil

**DOI:** 10.1007/s42770-026-01887-y

**Published:** 2026-03-11

**Authors:** Luiza Curi Lemos, Brenda de Almeida Perret Magalhães, Luisa Dias Da Mota, Rossana Patrícia Basso, Carla Vitola Gonçalves, Ana Clara Araújo De Santana, Rubens Caurio Lobato, Ana Maria Barral De Martínez, Andrea Von Groll, Melissa Orzechowski Xavier, Ivy Bastos Ramis, Marcelo Alves Soares, Vanusa Pousada Da Hora

**Affiliations:** 1 Faculty of Medicine, Universidade Federal do Rio Grande, Rio Grande, RS Brazil; 2 Hospital Universitário Dr. Miguel Riet Corrêa Jr, Universidade Federal do Rio Grande, Rio Grande, RS Brazil; 3 Oncovirology Program, Brazilian National Cancer Institute (INCA), Rio de Janeiro, RJ Brazil; 4 Laboratório de Biologia Molecular, FAMED/FURG, Rua Visconde de Paranaguá, s/n, Centro, Rio Grande, RS 96200-190 Brazil

**Keywords:** COVID-19, Coronavirus, Human immunodeficiency Virus, Hospitalization, Comorbidities, Coinfection

## Abstract

The COVID-19 pandemic, caused by SARS-CoV-2, has resulted in millions of infections and deaths. Studies describing the pathophysiology and characterizing the outcome of the disease in specific populations, including those living with HIV, are necessary. There is no consensus on whether the disease worsens in immunosuppressed patients, which is considered a risk factor, or if immunosuppression might confer protection against cytokine storms. This retrospective descriptive study reports on the profile of SARS-CoV-2/HIV-1 coinfection in individuals seeking care at the Miguel Riet Correa Jr. University Hospital, located in the southernmost part of Brazil, between January 2020 and December 2022.

During this period, 363 HIV-positive individuals sought diagnostic services for COVID-19, with 50 (13.7%) testing positive for the novel coronavirus. Among the 363 patients tested, the average age was 42.7 years, and 50.4% were female. For those with coinfection, several variables were analyzed, revealing a predominantly female population (62%), with 60% of white skin color and an average age of 47.8 years. Among these individuals, 48% exhibited symptoms characteristic of COVID-19, 40% had some comorbidity or coinfection, and 32% required hospitalization. Among the hospitalized patients who were discharged, 55% had discontinued HIV treatment, and among those not adhering to antiretroviral therapy (ART), 66.7% died from COVID-19.

The average hospital stay observed was 24 days, and 41.2% of patients required supplementary oxygen. Conversely, among patients who did not require hospitalization, 95% were on ART. These findings are consistent with other studies, suggesting that the course of COVID-19 in people living with HIV is similar to that in the general population, with attention to comorbidities as a risk factor in this group. It may be suggested that maintaining HIV treatment adherence could be protective against COVID-19 hospitalization (*p* = 0.002). Prevalence testing and Chi-square tests were used for data analysis to observe the correlation between hospitalization and discharge outcomes when considering the use of antiretroviral therapy for HIV treatment.

## Introduction

SARS-CoV-2 was the etiological agent responsible for the COVID-19 pandemic. Since 2019, there have been accounted more than 700 million cases and 7 million deaths worldwide. Of this extraordinary number, Brazil represented 38 million cases and more than 708,000 deaths. In the south of Brazil, more than 8 million infections and 112,000 deaths have already been reported [1].

It is known that COVID-19 can aggravate, becoming a severe acute respiratory syndrome (SARS) in specific cases, especially in people with comorbidities [2]. The comorbidities cited as of increased risk for COVID-19 are diabetes mellitus, hypertension, obesity, chronic obstructive pulmonary disease (COPD), asthma, cardiovascular diseases, liver diseases and kidney diseases. These are related to an immunological based syndrome referred to as “cytokine storm”, a state of hyper-inflammation associated with greater severity of the patient’s condition and, therefore, a worse prognosis. This is due to the ability of SARS-CoV-2 to modulate the host’s immune system through several mechanisms, leading to irregular type I IFN responses and excessive inflammation [3–5].

Regarding the presence of HIV concomitant with SARS-CoV-2 infection, there is no consensus in the literature whether the former influences the likelihood of a patient to develop COVID-19. However, once a person living with HIV/AIDS is infected with SARS-CoV − 2, there is a greater chance of a long course of illness, difficult treatment, and higher mortality [6]. According to the CDC, if a person with HIV infection receives a diagnosis of at least one of a set of opportunistic illnesses or has laboratory values indicating advanced disease, his or her disease is classified as HIV Stage 3 (AIDS) [7].

Several characteristics of patients co-infected with HIV and SARS-CoV-2 have been reported, as mentioned in cohorts from Italy, the United States, and Spain [8–11]. However, the influence of SARS-CoV-2 infection on people living with HIV is unclear. These studies aimed to determine whether COVID-19 worsens in patients living with HIV, or whether HIV acts as a protective factor against the immune system’s exacerbated response to SARS-CoV-2 [12].

According to the CDC [13], patients living with HIV could have a greater chance of developing more serious cases of the disease caused by the new coronavirus. Corroborating this fact, some studies have demonstrated the importance and influence of SARS-CoV-2 infection in individuals living with HIV, since the risk of developing, being hospitalized, and dying from COVID-19 was higher in this population than in those individuals who do not live with HIV [14, 15]. In contrast, data from a Spanish cohort [9] suggest that living with HIV would not aggravate COVID-19, but rather the relatively high presence of comorbidities in these people, and the use of antiretrovirals could be a factor in mitigating the severity of the disease.

Some cohort studies explored the outcome of SARS-CoV-2/HIV co-infection, elucidating the clinical features of COVID-19. An Italian cohort [16] reported the co-infection in 55 patients, identifying the presence of symptoms such as cough, fever, and dyspnea and the presence of asymptomatic cases. In this same study, the presence of comorbidities was associated with the need for mechanical ventilation. Furthermore, the authors describe that age over 50 years was associated with the need for oxygen supplementation and age over 60 years with the need for invasive treatment.

Of those patients whose outcome was death, all had comorbidities and advanced age. A Chinese cohort [2] described the characteristics of COVID-19 in 35 people living with HIV, of which 15 had severe illness and 2 died. Risk factors associated with the disease, age over 50 years, and discontinuation of antiretroviral treatment were highlighted. An American cohort [11] presented COVID-19 in 88 patients living with HIV, noting that the average age of the group was 61 years old, with a higher rate of black and Hispanic/Latino people and a high number of smokers and people with comorbidities.

A more robust study conducted in the United Kingdom analyzed data from 17 million individuals regarding the cause of death due to SARS-CoV-2 infection, showing that 25 deaths occurred in people living with HIV, with an estimated mortality of 0.087% in this group, and an estimated mortality of 0.038% in people without HIV, concluding that people with this cause of immunosuppression would be more likely to the outcome “death from COVID-19” when compared to the group without HIV [8]. Therefore, studies that describe the outcome of SARS-CoV-2/HIV co-infection are necessary, to elucidate the course of COVID-19 in this group which has an increased risk for many other diseases. This study aimed to characterize the course of COVID-19 in people living with HIV treated at a reference hospital for the treatment and diagnosis of HIV/AIDS in the extreme south of Brazil.

## Materials and methods

In this study, we evaluated the outcome of COVID-19 in patients living with HIV, treated in southern Brazil at the University Hospital Miguel Riet Correa Jr, part of the Federal University of Rio Grande (HU-FURG/EBSERH). All patients who presented co-infection between June 2020 and March 2022, not vaccinated against COVID-19, over 18 years of age, and exempt from informed consent, as approved by the HU-FURG ethical committee approval number 5.501.796, were included.

Our database was created using data available from the hospital’s physical and electronic medical records, comprising epidemiological data such as age, gender, marital status, level of education, skin color, presence of comorbidities and symptoms during infection, HIV viral load, CD4 T lymphocyte count, and cycle threshold (Ct) of real-time RT-PCR for SARS-CoV-2.

According to the CDC, there are several underlying clinical conditions that increase the risk of worsening the COVID-19 situation; that is, certain diseases can cause a more complex interaction with COVID-19 in individuals [17, 18]. Additionally, non-communicable diseases such as cardiovascular and metabolic comorbidities or malignancies are more prevalent in PLWH compared to those without HIV, thereby adding risk factors for the severity of COVID-19 [19]. Based on this, the present study considered the following comorbidities in the studied population: diabetes, hypertension, obesity, chronic obstructive pulmonary disease, severe asthma, cancer, and diseases associated with the immunosuppressive state of AIDS patients.

PLWH seem to exhibit the same clinical presentation of severe COVID-19 as the general population, although it is necessary to consider their virological and immunological status. Despite the clinical presentation apparently not differing significantly, there are some opportunistic infections that can mimic or coexist with COVID-19 [20].

Regarding the likelihood of hospitalization due to COVID-19 in PLWH, some studies have found no differences compared to people without HIV, with no increased hospitalization rates in this population [21, 22]. In contrast, the results of a meta-analysis of six studies showed that PLWH are indeed more likely to be hospitalized due to COVID-19 compared to people without HIV [23].

Thus, to estimate the extent of the consequences triggered by COVID-19, the following aspects were investigated in these patients:

1. disease symptoms similar to those of influenza and Severe Acute Respiratory Syndrome (SARS);
2. need for hospitalization;
3. extent of hospitalization;
4. need for supplemental oxygen (O2) during hospitalization;
5. COVID-19 outcomes; and.
6. use of antiretroviral therapy (ART) for HIV treatment.

Despite our small sample size, a descriptive rate (prevalence) for the variables was analyzed using SPSS version 20.0. In addition, we used the Chi-Square test to observe the correlation between hospitalization and discharge outcomes when using an antiretroviral regimen for HIV treatment. More advanced statistical analyses were tested but did not reach statistical significance.

## Results and discussion

During the research period, 363 people living with HIV underwent investigation for SARS-CoV-2 infection at the HU-FURG/EBSERH hospital. Of those, 13.7% (50/363) presented co-infection between these viruses. Among all 363 patients tested, the mean age was 42.7 years (SD ± 12.4), and 50.4% were female.

**Fig. 1:**
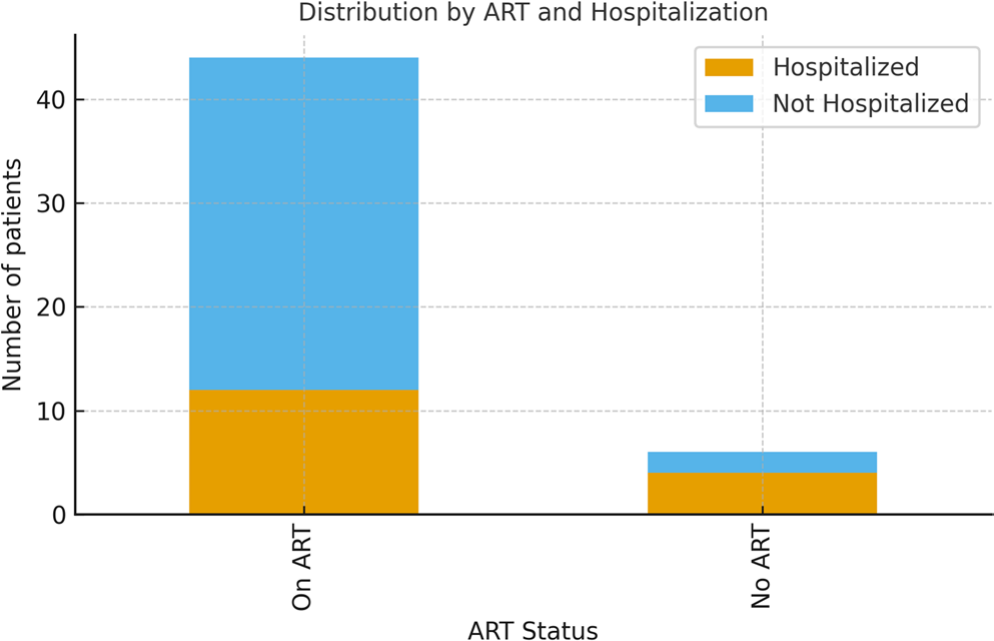
Distribution of patients by art status and hospitalization outcomE

Regarding sociodemographic variables, this study investigated: sex; average age; skin color; level of education; symptoms; comorbidities; need for hospital admission; abandonment of HIV treatment; outcome of COVID-19; average HIV viral load; mean CD4 T lymphocyte count and mean real-time RT-PCR Ct values for SARS-CoV-2 diagnosis. Of these variables, statistical significance was observed regarding to HIV antiretroviral treatment and hospitalization, with individuals receiving treatment having a lower chance of hospitalization. Figure 1 shows the distribution of patients by ART status and hospitalization outcome.

These variables were chosen because some research correlates the effect of advanced age on disease mortality and, interestingly, ethnicity. As an example, a cohort study from the United Kingdom demonstrated people living with HIV had a 2.9 times higher risk of death from COVID-19 compared to those without HIV, after adjusting for age and sex. Curiously, among people living with HIV, the risk of death was significantly higher for individuals of Black ethnicity compared to non-Black ethnicity [8]. Additionally, a Chinese study demonstrated that individuals who discontinued HIV treatment had a higher chance of having COVID-19 [2].

**Fig. 2:**
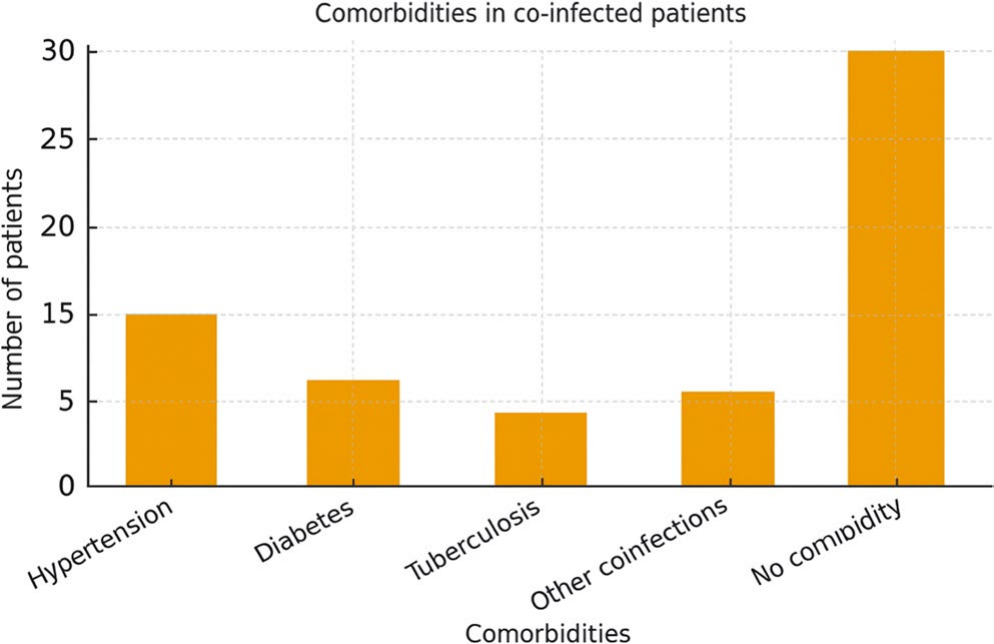
Distribution of comorbidities among co-infected patients

Among the 50 individuals with HIV/SARS-CoV-2 co-infection, 62% (31/50) were women, the average age was 47.8 years (SD ± 15.1), 60% (30/50) self-declared white and 50% (25/50) with complete primary education. Regarding symptoms, 48% (24/50) had some characteristic symptom of COVID-19, and subjects waited on average 2.28 days (SD ± 3.3) of symptoms before taking the test. 40% (20/50) had at least one comorbidity and/or co-infection. Figure 2 illustrates the distribution of comorbidities among co-infected patients.

Regarding ARV treatment of subjects, ten different protocols were used among HIV patients. The most prevalent protocol was Tenofovir Disoproxil Fumarate (TDF) + Lamivudine (3TC) + Dolutegravir (DTG) regimen, administered to 34% of patients (17/50). We observed that 14% of patients (7/50) were not under treatment for HIV at the time of SARS-CoV-2 diagnosis. With respect to need for hospitalization, 32% of patients (16/50) were hospitalized, with an average number of days in hospital facilities of 24 days (SD ± 17.7). Of these patients, 41.2% (7/16) required the use of supplemental oxygen, 75% (12/16) were discharged, three died and one outcome was unavailable.

The mean CD4 T lymphocyte counts in the hospitalized group was 462.50 cells/mm³ (SD ± 347.4), the mean HIV viral load was 25,288.87 copies/mL (SD ± 99,456.14), and the average Ct of the SARS-CoV-2 E gene at diagnosis was 29.59 (SD ± 7.7). Among the 16 patients who required hospitalization for treatment of COVID-19, 10 had clinical AIDS.

In a correlation analysis between the study variables using the Pearson’s Chi-Square test, we observed that 55% of the patients who were hospitalized and discharged had abandoned treatment for HIV, and among those who died from COVID-19, 66.7% were not using ARV. However, among patients who were not hospitalized, 95% were under HIV treatment (*p* = 0.002).Data regarding COVID-19 in patients who required hospitalization can be individually seen in Table 1.

**Table 1.** Simplified data on hospitalized sars-cov-2/hiv-1 co-infected patients

Patient Age	Days Hosp.	Symptoms	O2 Needed	Outcome	CD4 (cells/mm³)	ART Status
34	60	Asymptomatic	No	Discharge	80	No treatment
44	27	Symptomatic	Yes	Discharge	113	No treatment
70	3	Symptomatic	Yes	Death	317	On ART
24	2	Symptomatic	No	Discharge	763	On ART

In a bibliographic search covering the three years resulting from the COVID-19 pandemic, it was possible to observe studies addressing the outcome of SARS-CoV-2/HIV co-infection in different countries. Sample numbers similar to that of the present study were reported, as described in an Italian study (*n* = 47) and a Mexican study [24], which reported data from 55 patients.

With respect to gender, patients in this study were predominantly female, which corroborates the results evidenced in a New York cohort that demonstrated a high incidence of hospitalization and death from COVID-19 in women living with HIV when compared to men [25]. These data oppose what was found in other studies, such as the Spanish [26] and Chinese groups [2], where the majority of co-infected people were men. The predominance of women in our sample reflects the profile of PLWHIV seeking care at HU-FURG/EBSERH, mostly composed of women.

60% of the patients in this study were white, which agrees with what was presented in an Italian research [27]. On the other hand, other studies suggest that a large part of the population that presented HIV/SARS-CoV-2 co-infection was of black ethnicity [8, 11, 28]. However, these data may vary according to the region studied. In Rio Grande/RS, where this study was carried out, 79% of the population self-declares as white, according to the State Government of Rio Grande do Sul.

The average age of the participants in this study was a little over 47 years old, similar to what was mentioned by the Italian group [27], and lower than a London study [29] that reported an average age of 63 years. The average age of people with COVID-19 in the general population is over 60 years-old [30]. This difference can be explained by evidence suggesting that HIV infection accelerates biological aging, adding almost 10 years to chronological age.

**Fig. 3:**
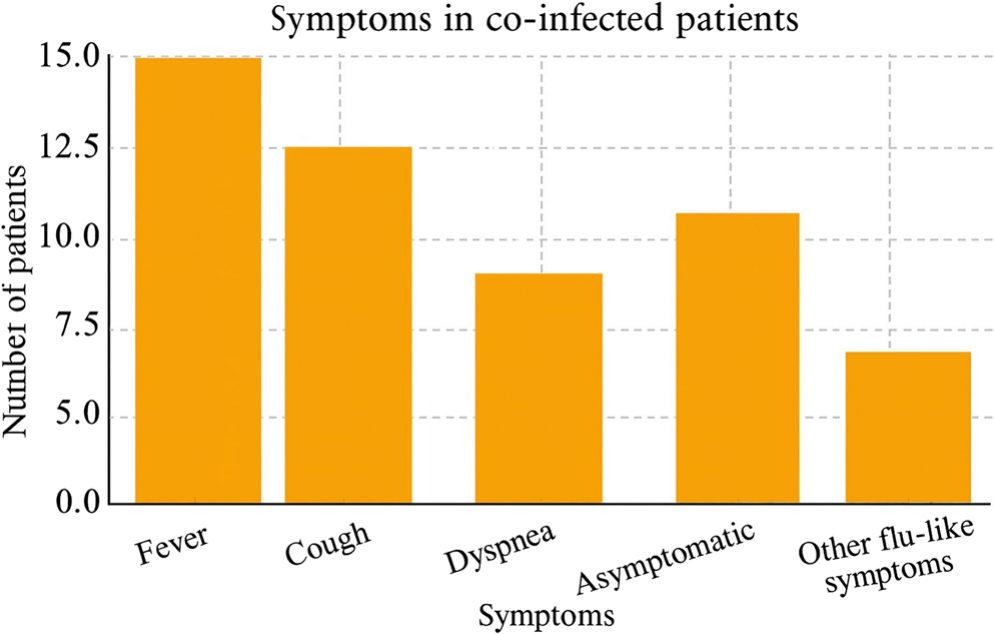
Frequency of symptoms reported by co-infected patients

Regarding the presence of comorbidities, 40% of individuals had some condition considered a risk factor for COVID-19 aggravation. Symptoms described were consistent with those of the general population (flu-like symptoms and, in severe cases, SARS) [2]. Figure 3 summarizes the frequency of symptoms reported by co-infected patients.

Among the patients included in this study, we observed that those who did not require hospitalization had satisfactory adherence to HIV treatment (60% of individuals were under current ARV schemes). A Chinese cohort identified that people discontinuing HIV treatment were at a greater risk for COVID-19 [2]. In our study, it was possible to associate that not abandoning HIV treatment was a protective factor against hospitalization for COVID-19. WHO Global Clinical Platform data reinforce that ART adherence and viral suppression reduce the risk of severe outcomes among co-infected patients.

When observing those patients who required hospitalization for the treatment of COVID-19, the average days of hospitalization were 24 days, differing from what a robust Spanish cohort [9] of 236 SARS-CoV-2/HIV patients showed, with average days of hospitalization of 7 days, and from an Italian case series [27] with 9.2 days. This prolonged hospitalization may be explained by the presence of multiple co-infections and advanced immunosuppression among our patients. The presence of different infections concomitantly with COVID-19 was also observed, such as histoplasmosis, tuberculosis, infection with *Klebsiella pneumoniae* and resistant *Candida* spp strains, diabetes, hypertension, and anemia, as well as a history of previous infections that left sequelae, such as neurosyphilis. These coexisting conditions likely contributed to the complexity of management and prolonged hospital stay.

A limitation of the study concerning the small sample size of the casuistics is recognized. Another limitation was the impossibility of comparing coinfected patients with a non-HIV group treated at the same center during the same period, due to database restrictions. Nevertheless, it is noteworthy that COVID-19 did not appear to be more severe in PLWH, even in hospitalized patients with multiple comorbidities or coinfections.

## Conclusions

This study demonstrated that the clinical outcome of COVID-19 in people living with HIV was similar to that observed in the general population. However, comorbidities and lack of adherence to ART were associated with worse outcomes, including hospitalization and death. It can be suggested that maintaining adherence to antiretroviral therapy may act as a protective factor against hospitalization for COVID-19 in this population. Although the number of cases in this study was limited, the findings highlight the importance of HIV treatment adherence and monitoring of comorbidities in patients with SARS-CoV-2/HIV-1 co-infection. Further multicenter studies with larger sample sizes are needed to confirm these observations and provide broader evidence.

## Data Availability

The authors confirm that all data underlying the findings are fully available without restriction.
